# Determination of hydrogen cyanide concentration in mainstream smoke of tobacco products by polarography

**DOI:** 10.1186/s40201-015-0211-1

**Published:** 2015-07-29

**Authors:** Shabnam Mahernia, Arash Amanlou, Gita kiaee, Massoud Amanlou

**Affiliations:** Department of Medicinal Chemistry, Faculty of Pharmacy and Pharmaceutical Sciences Research Center, Tehran University of Medical Sciences, Tehran, Iran

**Keywords:** Tobacco products, Cigarette, Hydrogen cyanide, Polarography

## Abstract

**Background:**

There has been a worldwide concern for the health risks of cigarette smoking and hydrogen cyanide (HCN) considered as one of the hazardous tobacco compounds which is needed to be determined in order to reduce the dose related to smoke disease risk.

In this study, we prepare the experimental procedure to entrap the HCN from mainstream smoke of different brands of Tehran cigarette, through simulating human inhalation and determine its concentration applying polarography.

**Results:**

The HCN level of the 50 commonly consumed tobacco products (47 cigarettes and 3 cigars) obtained from local store is ranged between 17.56 ± 1.02 and 1553.98 ± 0.56 μg per stick, this acquired amount is more than FDA approval (10 μg per stick), so the harmful effects of smoking is indicative.

**Conclusions:**

The comparative study of the results shows that the price and the weight of each product do not indicate HCN level. As can be seen, R^2^ value which is a statistical measure of how close the data are to the fitted regression line is low (R^2^ < 0.2). So it should not be deceived by names such as ultra light or infinite gravity to suck, because this names or the price haven^’^t effect on the amount of HCN and its destructive effects.

## Background

Smoking is the major risk factor of mortality in the world according to the statistical information; the cigarette consumption during one century has increased over 100 times, which increases the concern over the safety of tobacco products [1, 2].

Tobacco smoke contains more than 5000 chemical compounds which 150 of these substances have been proved to be toxicants [3, 4]. Hydrogen cyanide is one of the tobaccos smoke poisonous substances which are formed from the combustion of the protein and nitrate compounds existed in tobacco at high temperatures in the oxygen deficient condition [5, 6] which it’s chronic and low exposure causes neurological, respiratory, cardiovascular and thyroid effect [7–9].

The tobacco smoke pathway includes the part directly entered the mouth called mainstream and the part diffused in the surrounding, called side stream, which hazardous for nonsmoker. The level of HCN in mainstream smoke is ranged from 10 to 400 μg per cigarette (US Brands) which 0.6 to 27 % of these amounts exist in side stream smoke [10]. In the previous study the level of HCN in the non-filtered cigarette was between 400 to 500 μg per stick [11] and in another study the amount of HCN in the mainstream smoke of cigar, non-filtered cigarette and filtered cigarette was 1035, 59 and 448 μg per 1 g of tobacco, respectively and in small cigars it was between 510 to 780 μg per 1 g of tobacco [12].

There are different reported methods determine HCN in different samples including voltammetry [13], fluorometry [14, 15] gas chromatography [16], LC-MS-MS [17], HPLC-MS [18], potentiometry [19], spectrophotometry [20] and colorimetry [21]. Among them, polarography the subclass of voltammetry is the most precise and inexpensive method based on the oxidation and reduction mechanism [22].

The aim of this study is focused on the determination of the HCN in mainstream smock of different brands of cigarette consumed in Tehran using polarography method.

## Materials and methods

### Collection of samples

A total of 50 types of different brands of the most consumed or available cigarettes (47) and cigars (3) were collected from local stores at September 2012. The 20 % of the samples were from Winston company, 18 % from Marlboro, 6 % from Magna, 8 % from KENT and 8 % from local or officially imported companies and the rest, 40 % are from a different companies (as shown in Table 1).

**Table 1:** Hydrogen cyanide concentration and price of different brands of cigars

No.	Brand	Cyanide concentration (μg/cig.)	Number of cig. per pocket	The price of each pocket($)	Weight of each cig. (mg)	The price of each cig. ($)
1	Bahman	368.067	20	0.58	828.4	0.02
2	Bahman	574.358	20	0.50	587.7	0.02
3	Bahman	57.655	20	0.50	547.8	0.02
4	Bistoon	227.491	20	0.41	943.7	0.02
5	CAFÉ CRÈME	791.067	10	2.08	921.6	0.20
6	CAFÉ CRÈME (AROME)	1464.900	10	2.08	1053.2	0.20
7	CAFÉ CRÈME (BLUE)	406.722	10	1.83	746.6	0.18
8	CAMEL Lights	184.825	20	1.62	810.1	0.08
9	Cima	237.400	20	0.66	863.9	0.03
10	Cima classic	120.720	20	0.60	687.6	0.03
11	Dunhill	300.717	20	2.25	781.9	0.11
12	Eclipse	740.235	20	12.50	1243.6	0.62
13	ESSE Lights	131.012	20	0.80	515.2	0.04
14	ESSE Special Gold	29.622	20	0.66	530.2	0.03
15	Jewels sweet	1553.584	20	1.62	6165.5	0.08
16	Kent	297.693	20	1.33	793.7	0.06
17	Kent	190.745	20	1.25	436.9	0.06
18	Kent (Blue7)	345.930	20	1.25	915.8	0.06
19	Kent (White1)	188.220	20	1.25	776.2	0.06
20	King Edward	598.518	5	1.41	3384.2	0.28
21	Magna	232.889	20	0.79	822.4	0.03
22	Magna	145.228	20	1.79	763.8	0.08
23	Magna	212.310	20	0.79	813.3	0.04
24	Marlboro Gold (Germany)	165.871	20	3.75	800.3	0.18
25	Marlboro (Extra) (USA)	164.309	20	4.16	900.2	0.20
26	Marlboro (Switzerland)	17.561	20	2.66	849.1	0.13
27	Marlboro (Switzerland)	47.892	20	2.91	969.7	0.14
28	Marlboro Lights	267.804	20	2.50	858.9	0.12
29	Marlboro Lights (USA)	42.916	20	2.91	776.4	0.14
30	Marlboro Lights (Switzerland)	74.536	20	2.91	786.5	0.14
31	Marlboro Lights (Switzerland)	69.344	20	1.83	816.5	0.09
32	Marlboro Ultra Lights (Switzerland)	127.336	20	2.66	835.1	0.13
33	Montana	332.493	20	0.50	855.6	0.02
34	Pall Mall	161.785	20	0.83	905.1	0.04
35	Pall Mall (Blue)	86.956	20	0.83	881.3	0.04
36	PHILLIES BLUNT	203.178	5	1.62	6974.5	0.32
37	Pine (Blue)	238.561	20	0.66	860.2	0.03
38	Pine (supper slims)	94.813	20	0.45	547.2	0.02
39	Winston	43.187	20	1.25	785.9	0.06
40	Winston	214.325	20	1.50	816.7	0.07
41	Winston	106.176	20	1.50	831.0	0.07
42	Winston Blue (Europe)	99.244	20	1.83	537.3	0.09
43	Winston Lights	66.326	20	2.50	943.1	0.12
44	Winston Lights (Imported)	209.294	20	4.58	814.0	0.22
45	Winston Lights (USA)	102.132	20	2.08	808.7	0.10
46	Winston Ultra Lights (USA)	42.634	20	2.25	794.1	0.11
47	Winston Ultra Lights (USA)	25.554	20	2.25	818.4	0.11
48	Winston Ultra Lights (Switzerland)	254.322	20	2.25	812.0	0.67
49	Zest Lights	150.623	20	0.66	829.9	0.03
50	Zika	288.120	20	2.91	924.0	0.14

### Reagents and chemicals

All chemicals used were of analytical reagent grade from Merck (Germany). Buffer solutions were prepared by dissolving boric acid (0.2 M) and potassium hydroxide (0.17 M) in 1000 ml ultrapure water and adjusting the solution to pH 10.2. Cyanide standard solution (1 g/L) was prepared by dissolving 0.2503 g KCN in 100 ml KOH 0.01 M in ultrapure water.

### Apparatus

Analysis was conducted by the Metrohm Polarography device 797 VA Computrace, three electrode systems consisting of a dropping mercury electrode (DME) as the working electrode, an Ag/AgCl reference electrode and platinum counter electrode. The device outfitted in the following conditions: stirrer speed 2000 rpm, mode DP, purge time 300 s, equilibration time 5 s, pulse amplitude 50 mV, start potential 0 V, end potential −500 mV, voltage step 8 mV, voltage step time 0.8 s, sweep rate 10 mV/s, peak potential CN −240 mV. All instrumental settings were those recommended in the manufacturer’s manual book and the instrumental conditions with the method of AB110-Det of cyanide [23]. All potentials quoted were measured against an Ag/AgCl reference electrode and the polarographic cell volume was 20 ml.

### Method of analysis

**Fig. 1:**
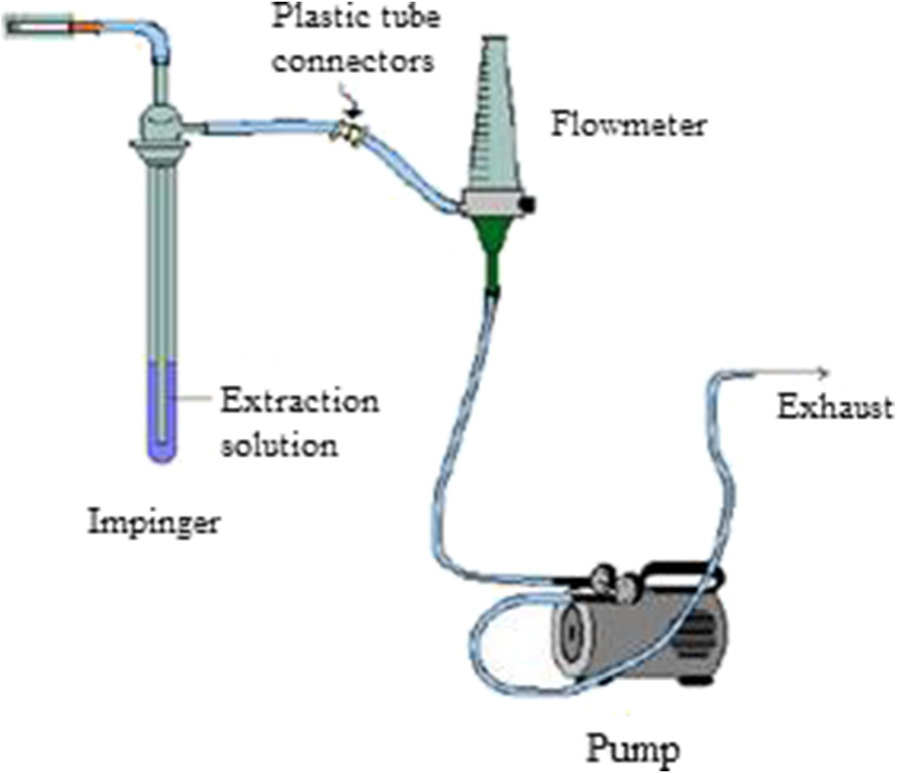
Cigarette mainstream collecting apparatus

The HCN in each cigarette and cigar mainstream smoke were collected using mainstream apparatus (Fig. 1) [24, 25]. The cigarette was applied to the entrance station and suction force obtained by the vacuum pump simulate the human inhalation and extract the tobacco smoke, the flow of smoke were passed through the glass tube filled with 100 ml NaOH (0.1 M) solution to entrap HCN as CN^−^ ion.

**Fig. 2:**
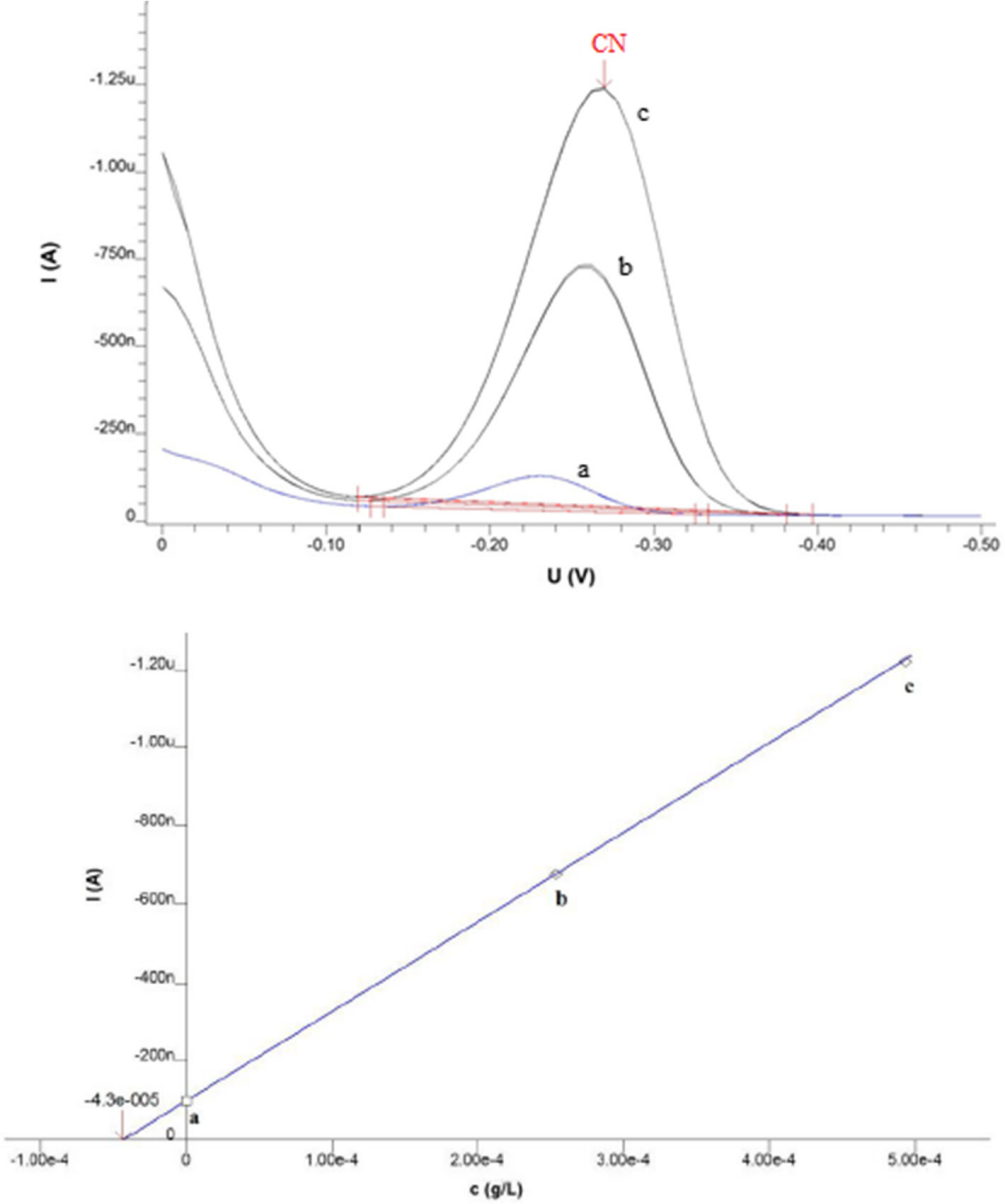
The differential pulse polarogram of cyanide ion in cigarette smoke sample. **a** the peak of sample solution **b** the peak of first dilution of standard addition **c** the peak of second dilution of standard addition

Subsequently, the mixture of above mentioned solution contain cigarette smoke (10 ml) and buffer solution (10 ml) was added to the polarographic vessel and deoxygenated for 10 min with high-purity nitrogen and achieved the peak of CN^−^ in the range of −0.5-0 V. To determine the CN^−^ concentration by standard addition method, 50 μl cyanide standard solution was added (two times) and the polarogram was obtained (Fig. 2).

### Statistical analysis

All statistical analyses were performed using the statistical software for social sciences (SPSS Inc. Chicago, IL, Version 21). Statistical analyses of each sample were characterized by mean ± standard deviation. The mean levels of cyanide were compared across categories of price, and weight of each cigarette or cigars. The significance level was defined at 0.05 for the regression equations.

## Result and discussion

The result of mainstream smoke HCN determination by polarography (Table 1) showed that among 50 samples the average amount of HCN was 184.825 μg per stick which the highest level of HCN pertain to Jewel sweet cigar, 1553.98 ± 0.56 μg per cigar, and the lowest was Swiss Marlboro with 17.56 ± 1.02 μg per cigarette. Moreover, the average amount of HCN in cigarettes and cigar were 218 μg and 785.09 μg, respectively, In addition the average weight of cigarette and cigar samples was 800 mg and 5500 mg, respectively, and the average weight of whole samples was 1084.24 mg.

The changes in the HCN levels are dependent on the preparation procedure of tobacco, which lead to the diverse amount of proteins and nitrate compounds of cigarette which render the conversion of HCN level emission. In addition it may be possible to assess the quality of cigarette especially ultralight by determining the amount of HCN as it is increased in the unfeigned products.

**Fig. 3:**
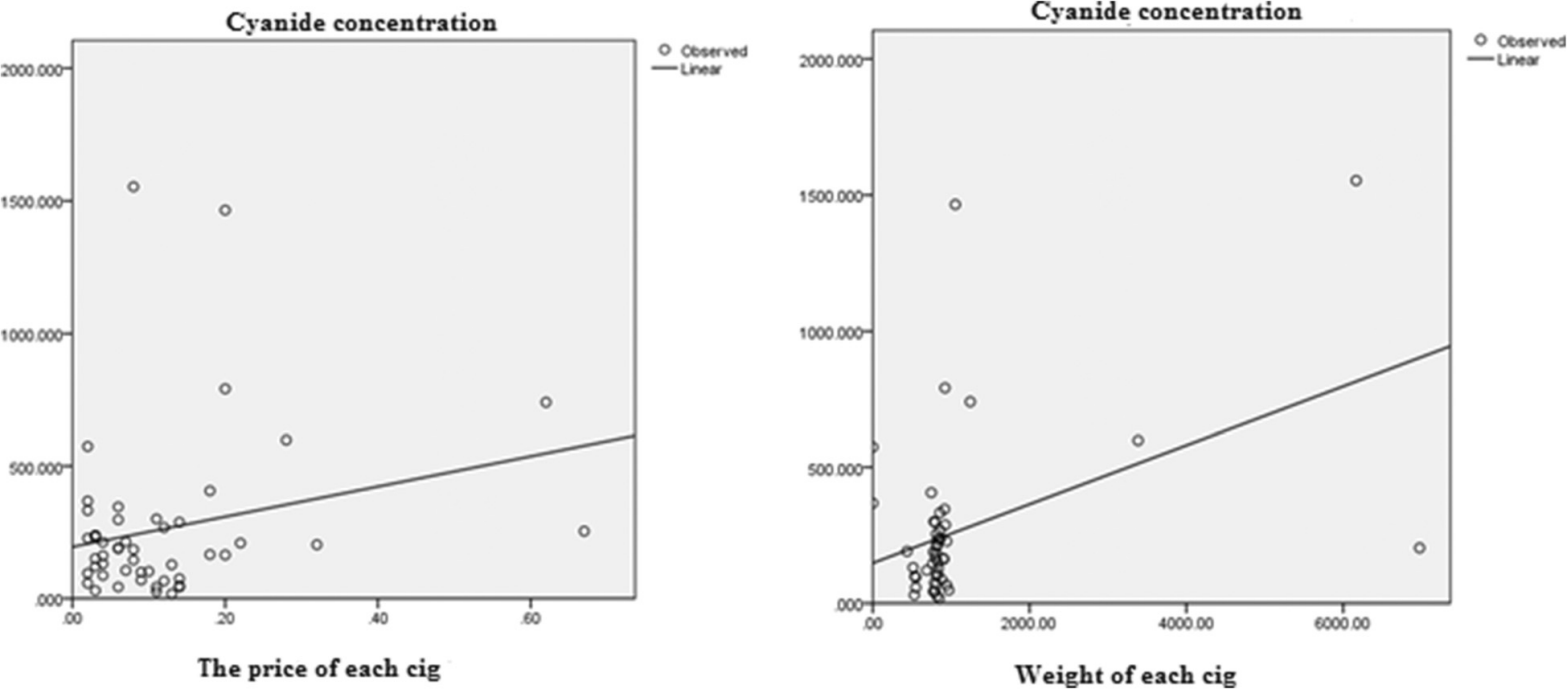
The relationship between cyanide, weight and price each of cigarettes

Furthermore, based on our study there is no connection between price, weight and appearance of cigarette, cigar and HCN level. For instance, Jewels sweet and PHILLIES BLUNT cigars have the same price, but different level of HCN doesn’t necessarily cause the higher HCN level emission. The regression method was applied for the development of the mentioned model. Validation of the method was conducted by analyzing the different set of independent data from the same source. As can be seen the R^2^ which is a statistical measure of how close the data are to the fitted regression line is low (0.20) (Fig. 3). So it means the model is useless for prediction based on cigar and cigarette weight and its price.

**Fig. 4:**
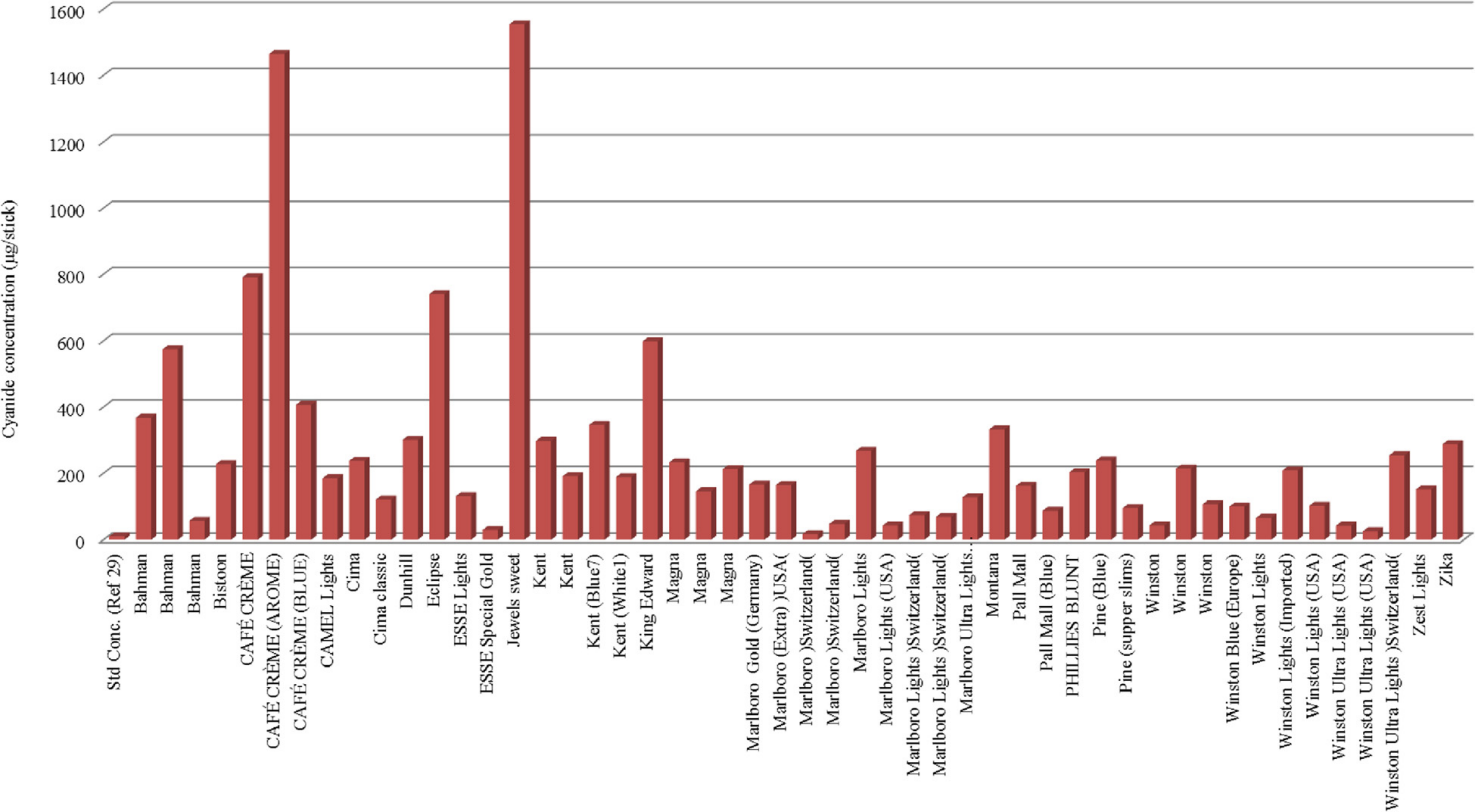
Cyanide concentrations in different kind of Cig

In addition, previous studies demonstrated that even exposure to lower concentrations of cyanide may result in a range of non-specific features include headache, dizziness, throat discomfort, chest tightness and eye irritation which these symptoms would grow by more substantial exposure [26–28]. Moreover the results of our study indicate that cyanide concentration of all samples, even light cigarette is above established levels for chronic toxic doses [9] (Fig. 4).

## Conclusions

In this study, HCN of the toxic components of the tobacco smoke was determined by polarographic method. The results shows that the ranges of HCN level of 50 samples were varied between 17.56 ± 1.02 - 1553.98 ± 0.56 μg per stick. This acquired amount is more than FDA approval (10 μg per stick).

## References

[1] General S (1989). Reducing the health consequences of smoking. 25 years of progress. US Department of Health and Human Services.

[2] Shafey O, Eriksen M, Ross H, Mackay J (2009). The Tobacco Atlas.

[3] Fowles J, Dybing E (2003). Application of toxicological risk assessment principles to the chemical constituents of cigarette smoke. Tob Control.

[4] Green CR, Schumacher JN, Llovd RA, Rodgman A (2007). Comparisons of the composition of tobacco smoke and the smokes from various tobacco substitutes. Beit Tabakforsch Intl.

[5] Norman V, Ihrig A, Larson T, Moss B (1983). The effect of some nitrogenous blend components on NO/NOx and HCN levels in mainstream and sidestream smoke. Beitr Tabakforsch.

[6] Baker R (1999). Smoke Chemistry, Tobacco production.

[7] Blanc P, Hogan M, Mallin K, Hryhorczuk D, Hessl S, Bernard B (1985). Cyanide intoxication among silver-reclaiming workers. JAMA.

[8] Chandra H, Gupta B, Bhargava S, Clerk S, Mahendra P (1980). Chronic cyanide exposure biochemical and industrial hygiene study. J Anal Toxicol.

[9] El Ghawabi SH, Gaafar MA, El-Saharti AA, Ahmed SH, Malash KK, Fares R (1975). Chronic cyanide exposure. a clinical, radioisotope, and laboratory study. Br J Ind Med.

[10] Fiksel J, Slimak MW, Little AD (1981). An Exposure and Risk Assessment for Cyanide. Monitoring and Data Support Division, Office of Water Regulations and Standards.

[11] Hoffmann D, Hoffmann I. Risks of tobacco smoke exposure are similar for all sources of tobacco smoke, and the magnitude of the risks experienced by cigar smokers is proportionate to the nature and intensity of their exposure. Chem and Toxicol. 1998;55.

[12] Adams PI (1968). Combustion temperatures in cigars and cigarettes, A comparative study. Tobacco Sci.

[13] Safavi A, Maleki N, Shahbaazi H (2004). Indirect determination of cyanide ion and hydrogen cyanide by adsorptive stripping voltammetry at a mercury electrode. Anal Chim Acta.

[14] Lundquist P, Rosling H, Sörbo B, Tibbling L (1987). Cyanide concentrations in blood after cigarette smoking, as determined by a sensitive fluorimetric method. Clin Chem.

[15] Chen D, Castro M, Valcarcel M (1990). Flow-through sensor for the fluorimetric determination of cyanide. Talanta.

[16] Brunnemann KD, Yu L, Hoffmann D (1977). Chemical studies on tobacco smoke. XLIX. Gas chromatographic determination of hydrogen cyanide and cyanogen in tobacco smoke. J Anal Toxicol.

[17] Lacroix C, Saussereau E, Boulanger F, Goulle J (2011). Online liquid chromatography-tandem mass spectrometry cyanide determination in blood. J Anal Toxicol.

[18] Tracqui A, Raul J, Geraut A, Berthelon L, Ludes B (2002). Determination of blood cyanide by HPLC-MS. J Anal Toxicol.

[19] Bark L, Higson H (1963). A review of the methods available for the detection and determination of small amounts of cyanide. Analyst.

[20] Surleva AR, Visualizing DG, Hazard S (2013). A simple spectrophotometric determination of hydrogen cyanide in cigarette smoke and filters. J Chem Edu.

[21] Fowler E, Steele TW (1968). The determination of anionic surface-active agents in dilute aqueous solution.

[22] Surleva A (2009). Recent achievements in toxic cyanide monitoring: review. Revue electronique internationale pour la science et la technologie. Numéro.

[23] Polarographic determination of free cyanide No, 110/2 e. In: Metrohm application bulletin

[24] Determination of Hydrogen Cyanide in Sidestream Tobacco Smoke (1999). Health Canada.

[25] Diekmann J, Biefel C, Rustemeier K (2002). Analysis of cigarette mainstream smoke for 1,1-dimethylhydrazine and vinyl acetate by gas chromatography–mass spectrometry. J Chromatogr Sci.

[26] National Poisons Information Service (NPIS) Hydrogen cyanide (2000). Toxbase®.

[27] Lam KK, Lau FL (2000). An incident of hydrogen cyanide poisning. Am J Emerg Med.

[28] Agency for Toxic Substances and Disease Registry (ATSDR) (2004). Draft Toxicological Profile for Cyanide.

